# Changes of serum IGF-1 and ET-1 levels in patients with osteoporosis and its clinical significance

**DOI:** 10.12669/pjms.35.3.84

**Published:** 2019

**Authors:** Lei Sun, Jin Su, Mingming Wang

**Affiliations:** 1 *Lei Sun, Department of Orthopedics, Binzhou People’s Hospital, Shandong, 256600, China*; 2 *Jin Su, Department of Orthopedics, Binzhou People’s Hospital, Shandong, 256600, China*; 3 *Mingming Wang, Department of Orthopedics, Binzhou People’s Hospital, Shandong, 256600, China*

**Keywords:** Osteoporosis, Insulin-like growth factor-1, Endothelin-1, Cytokines, Bone density

## Abstract

**Objective:**

To investigate the correlations of levels of serum insulin-like growth factor-1 (IGF-1) and endothelin-1 (ET-1) with cytokines including interleukin (IL)-18, IL-6 and high-sensitivity C-reactive protein (hs-CRP) and bone material density in patients with osteoporosis d.

**Methods:**

Eighty patients with osteoporosis who were treated in our hospital from April 2016 to October 2017 were selected as observation group, and 60 healthy elderly people who received physical examination in our hospital in the same period were selected as control group. The serum levels of IGF-1 and ET-1 were detected using enzyme-linked immunosorbent assay. The bone material density of the lumbar vertebra, tibial neck and Ward’s triangle of every research subject was measured using dual-energy x-rays absorptiometry. The correlations between variables were analyzed using Pearson correlation analysis.

**Results:**

The level of IGF-1 in the observation group was lower than that in the control group, and the level of ET-1 in the observation group was higher than that in the control group (P<0.05). The levels of interleukin (IL)-18, IL-6 and high-sensitivity C-reactive protein (hs-CRP) in the observation group were significantly higher than those in the control group (P<0.05). Bone mineral density of lumbar vertebra, tibial neck and Ward triangle in the observation group was significantly lower than that in the control group (P<0.05); the IGF-1 level of osteoporosis patients was negatively correlated with IL-18, IL-6, hs-CRP levels and positively correlated with bone mineral density; the ET-1 level was positively correlated with IL-18, IL-6, hs-CRP levels and negatively correlated with bone mineral density.

**Conclusion:**

Patients with osteoporosis have decreased level of IGF-1 but increased level of ET-1, and they are closely related to cytokines and bone mineral density and may participate in the pathogenesis of osteoporosis.

## INTRODUCTION

Osteoporosis, a systemic bone metabolic disease, is mainly induced by bone density reduction and bone microstructure destruction caused by the abnormal metabolism of calcium and phosphorus in the body.^1^,^2^ An epidemiological study shows that 223~333 among 10 thousand people may have osteoporosis.^3^ The incidence of osteoporosis is higher in middle-aged and elderly people or in people with endocrine hormone metabolism disorders.^4^ A foreign study show that about 15% of white people in their 50s and 70% of white people over the age of 80 are affected, and women are more common than men.^5^ The diagnostic results in developed countries indicated that 2%~8% of males and 9%~38% of females had osteoporosis.^6^ As population aging becomes more and more serious in China, the prevalence of osteoporosis is increasing year by year. Osteoporosis has become an important cause of fracture and even death of patients, but there is no effective method for clinical diagnosis of osteoporosis.

Insulin-like growth factor-1 (IGF-1) as a hormone similar to insulin in molecular structure plays an important role in the development of children and continues to have anabolic function in adults. IGF-1 is produced throughout life, with the highest production rate in adolescence and the lowest level in infants and the elderly. IGF-1 is the main mediator of growth hormone (GH). Growth hormone is produced in the anterior pituitary lobe, released into the bloodstream, and then stimulates the liver to produce IGF-1. Then IGF-1 stimulates the growth of the whole body and promotes the growth of almost every cell in the body.^7^ Endothelin-1 (ET-1), also known as pre-proendothelin-1 (PPET-1), is the strongest vasoconstrictor found so far. Metabolic disorders, vasoconstriction, myocardial ischemia and cell proliferation induced by ET-1 are common pathogenic factors of vascular injury-related diseases.^8^

Changes in biological factors can affect the loss of bone metabolism, the synthesis of bone collagen fibers or the structure of bone trabeculae, thereby increasing the risk of osteoporosis. IGF-1 as a member of growth factor family can promote the regeneration of collagenous fiber in intercellular substance components and the stablization of bone trabecula structure.^9^ ET-1, an relevant indicator of peroxidation damage, can promote the decomposition of collagenous fiber and cytoskeletal structure and the loss of sclerotin or calcium salt.^10^ In order to further reveal the relationship between different biological factors and patients’ disease condition, this study analyzed the changes of serum levels of IGF-1 and ET-1 in patients with osteoporosis who were treated in our hospital and explored their relationships with cytokines and bone density. This work aims to offer a reference for clinical diagnosis and treatment of osteoporosis.

IGF-1, as a growth factor with high content in bone, plays an important role in regulating osteoclast proliferation and differentiation, osteocyte function, bone metabolism, etc.^11^ IGF-1 may be closely related to the generating process of osteoporosis.^7^ ET-1, as a vasoconstrictor peptide, is closely related to the proliferation of vascular endothelial cells. A study has shown that estrogen level can affect the biological activity of ET-1 and lead to microcirculation vascular dysfunction.^8^

## METHODS

Eighty patients with osteoporosis who were treated in our hospital from April 2016 to October 2017 were randomly selected as the observation group using convenience sampling method. The patients all met the diagnostic criteria of osteoporosis recommended by WHO.^12^ Exclusion criteria included having endocrine system diseases such as thyroid, parathyroid and diabetes mellitus, having important organ dysfunction such as the heart, liver and kidney, having malignant tumor, and taking calcium, vitamin D, heparin and hormone drugs in recent 6 months. Sixty elderly healthy volunteers who aged 65 or above and received physical examination in our hospital in the same period were randomly selected as controls, and osteoporosis and other orthopaedic diseases were excluded; other exclusion criteria were the same with the observation group. There were 80 elders in the observation group, including 37 males and 43 females. The age of subjects in the observation group ranged from 65 to 81 years (average (71.3±5.6) years). There were 60 elders in the control group, including 26 males and 34 females. The age of subjects in the control group ranged from 65 to 79 years (average (70.3±5.4) years). There was no significant difference between the two groups in age, sex and other general clinical data. The study was approved by the ethics committee of the hospital, and all the subjects signed informed consent.

All subjects were required to stop taking food and water six hours before detection. On the morning of the 2nd day, 5 ml of fasting venous blood was collected and divided into two parts. The first part was centrifuged at 3000 r/min for 10 minutes after natural anticoagulation. The supernatant was collected, and the serum IGF-1 and ET-1 levels were detected using a ST-360 automatic multi-function microplate reader (Shanghai Kehua Company, China) and enzyme-linked immunosorbent assay. Human IGF-1 and ET-1 detection kits were purchased from Shanghai Changjin Biotechnology Co., Ltd., China). The second part was placed in a sodium citrate anticoagulant tube. The levels of interleukin (IL)-18, IL-6 and high-sensitivity C-reactive protein (hs-CRP) were detected by colloidal gold method according to specifications of kits (Shanghai Upper Bio-tech Pharma Co., Ltd., China). The bone material density of the lumbar vertebra, tibial neck and Ward’s triangle of every research subject was measured using dual-energy x-rays absorptiometry (GE, USA).

The differences of serum IGF-1 and ET-1 levels between the two groups were observed. Moreover the levels of cytokines and bone mineral density were compared between the two groups. The correlations of the levels of IGF-1 and ET-1 with cytokines and bone mineral density were analyzed.

SPSS version 21.0 was used for statistical analysis. Mean and standard deviations were used for quantitative data analysis. Serum levels of IGF-1, ET-1 and cytokines and bone mineral density were compared between the two groups using t test. Pearson correlation analysis was used to analyze the correlation between the levels of IGF-1 and ET-1 and cytokines and bone mineral density. P<0.05 indicated that the difference was statistically significant.

## RESULTS

The IGF level of patients in the observation group was significantly lower than that in the control group, but the ET-1 level was significantly higher than that in the control group; the difference had statistical significance (P<0.05, Table-I).

**Table-I:** Comparison of serum IGF-1 and ET-1 levels between subjects in the two groups.

	Observation group	Control group	t	P
IGF-1 (ng/mL)	162.23±10.27	255.67±15.76	-37.874	<0.05
ET-1 (pg/mL)	56.86±6.87	38.13±4.31	17.862	<0.05

The levels of IL-18, IL-6 and hs-CRP of the observation group were significantly higher than those of the control group, and the differences had statistical significance (P<0.05, Table-II). The bone mineral density of the lumbar vertebra, tibia neck and Ward triangle of the observation group was significantly lower than that of the control group (P<0.05, Table-III).

**Table-II:** Comparison of levels of cytokines between subjects in the two groups.

	Observation group	Control group	t	P
IL-18 (mg/L)	52.36±9.63	28.18±3.14	18.459	<0.05
IL-6 (mg/L)	18.34±5.25	7.67±2.12	14.627	<0.05
hs-CRP (mg/L)	10.29±3.15	5.13±1.17	11.819	<0.05

**Table-III:** Comparison of bone mineral density between subjects in the two groups.

	Observation group	Control group	t	P
Lumbar vertebra	0.78±0.18	0.93±0.25	-3.607	<0.05
Tibial neck	0.71±0.23	0.93±0.32	-4.396	<0.05
Ward’s triangle	0.72±0.26	0.92±0.31	-3.972	<0.05

Analysis on correlation of serum levels of IGF-1 and ET-1 with cytokines and bone mineral density showed that serum levels of IGF-1 were in a significant negative correlation with levels of IL-18, IL-6 and hs-CRP, but in a significant positive correlation with bone mineral density; the serum level of ET-1 was in a significant positive correlation with levels of IL-18, IL-6 and hs-CRP, but in a significant negative correlation with bone mineral density (Table-IV).

**Table-IV:** Correlation of levels of IGF-1 and ET-1 of patients in the observation group with cytokines and bone mineral density.

	r	P		r	P
IGF-1 vs IL-18	-0.613	<0.05	ET-1 vs IL-18	0.597	<0.05
IGF-1 vs IL-6	-0.626	<0.05	ET-1 vs IL-6	0.609	<0.05
IGF-1 vs hs-CRP	0.601	<0.05	ET-1 vs hs-CRP	0.622	<0.05
IGF-1 vs bone density	0.621	<0.05	ET-1 vs bone density	-0.611	<0.05

## DISCUSSION

This study found that the serum levels of IGF-1 and ET-1 had obvious abnormal expression in patients with osteoporosis, the expression level of IGF-1 was significantly decreased, and the expression level of ET-1 was significantly increased, indicating that different biological factors might participate in the occurrence and development of osteoporosis. IGF-1, a member of the growth factor family, has a structure similar to that of insulin. The content of IGF-1 in human skeleton is high.^13^ Therefore, it can help bone metabolism go on smoothly and promote the differentiation and proliferation of many kinds of cells, such as osteocytes. It can promote the re-establishment of vascular endothelium, interstitial cells or tissue cells in patients to improve the repair ability of patients’ organs.^14^ Xian et al. found that IGF-1 could inhibit bone resorption and promote bone formation.^15^ Moreover, IGF-1 can promote angiogenesis by accelerating the secretion of vascular endothelial growth factor in osteoblasts, which is the guarantee of bone growth.^16^ ET-1 which exists in osteoblasts, osteoblasts and osteoclasts is closely related to microcirculatory vasoconstriction function.^17^ ET-1 can inhibit the formation of new vessels, reduce the blood flow perfusion of bone mesenchymal component cells, induce the occurrence of hypoxic-ischemic oxidative stress dysfunction, and aggravate ischemia-reperfusion injury.^18^,^19^

The decreased expression of IGF-1 can affect bone mineral density and promote the occurrence of osteoporosis by affecting the repair of collagen fibers, the mechanical stress ability of bone trabeculae or the flow of interstitial components of bone cells.^20^ The increased expression of ET-1 can affect the clinical prognosis of osteoporosis through the following aspects. First, high expression of ET-1 can increase the increase amplitude of downstream cytokines such as IL-6 or IL-10, which can lead to a significant increase of cytokines in patients, promote the occurrence of local inflammation, and lead to further destruction of bone. Secondly, the increase of ET-1 can increase the risk of bone loss by activating oxidative stress, leading to disorder of calcium and phosphorus metabolism balance.^21^ Some researchers have discussed the changes of serum biological factor spectrum in elderly patients with osteoporosis and found that there were significant fluctuations in IGF-1 and other factors and IGF-1 decreased significantly with the progress of the disease and had obvious linear trend difference.^22^

IL-18, IL-6 and hs-CRP are risk factors for trabecular bone destruction in osteoporosis patients. Increased expression of related indicators in this study suggested the effect of inflammatory factors such as IL-18, IL-6 and hs-CRP on osteoporosis. Changes of bone mineral density in the observation group, especially the decrease in the lumbar spine, tibial neck and Ward triangle, indicates a significant increase in fracture risk. More importantly, this study found that IGF-1 and ET-1 were in a close correlation with cytokines or bone mineral density in osteoporosis patients, its internal mechanism was generally considered to be correlated to the increased release of inflammatory factors such as IL-18, IL-6 and hs-CR because of the activation effects of IGF-1 and ET-1 on monocyte or macrophage, which further indicated the relationship between the levels of IGF-1 and ET-1 and the condition of osteoporosis patients.

## CONCLUSION

The innovation of this study was to explore relationships of IGF-1, ET-1 with serum cytokines and bone mineral density in patients with osteoporosis. Osteoporosis patients have low level of IGF-1 and high level of ET-1, and IGF-1 and ET-1 are closely related to cytokines and bone mineral density.

### Authors’ Contribution

**LS:** Study design, data collection and analysis.

**LS & JS:** Manuscript preparation, drafting and revising.

**LS & MMW:** Review and final approval of manuscript.

## References

[1] Lin X, Xiong D, Peng YQ, Sheng ZF, Wu XY, Wu XP (2015). Epidemiology and management of osteoporosis in the People′s Republic of China:current perspectives. Clin Interv Aging.

[2] Swenne I, Stridsberg M (2015). Bone metabolism in adolescent girls with eating disorders and weight loss:independent effects of weight change, insulin-like growth factor-1 and oestradiol. Eat Weight Disord.

[3] Misof BM, Patsch JM, Roschger P, Muschitz C, Gamsjaeger S, Paschalis EP (2014). Intravenous treatment with ibandronate normalizes bone matrix mineralization and reduces cortical porosity after two years in male osteoporosis:a paired biopsy study. J Bone Miner Res.

[4] Zhang W, Zhang LC, Chen H, Tang PF, Zhang LH (2015). Association between polymorphisms in insulin-like growth factor-1 and risk of osteoporosis. Genet Mol Res.

[5] Gabriel SE, Kneeland TS, Moncur MM, Et-tinger B, Tosteson AN (1999). Health-related quality of life in economic evaluations for osteoporosis:whose values should we use. Med Decis Making.

[6] Khan A, Morrison A, Ruggiero S, Tetradis S, Davison KS, Peters E (2015). Response to comments on “diagnosis and management of osteoporosis of the jaw:A systematic review and international consensus”. J Bone Miner Res.

[7] Ashpole NM, Herron JC, Mitschelen MC, Farley JA, Logan S, Yan H (2016). IGF-1 regulates vertebral bone aging through sex-specific and time-dependent mechanisms. J Bone Miner Res.

[8] Horstmeyer A, Licht C, Scherr G, Eckes B, Krieg T (2010). Signalling and regulation of collagen I synthesis by ET-1 and TGF-beta1. Febs J.

[9] Liu R, Hu L, Sun A, Cao YJ, Tang T, Zhang XP (2014). mRNA expression of IGF-1 and IGF-1R in patients with colorectal adenocarcinoma and type 2 diabetes. Arch Med Res.

[10] Wang B (2008). Correlation between plasma endothelin level and bone mineral density in patients with postmenopausal osteoporosis. J Clin Rehab Tissue Eng Res.

[11] Yan J, Herzog JW, Tsang K, Brennan CA, Bower MA, Garrett WS (2016). Gut microbiota induce IGF-1 and promote bone formation and growth. Proc Natl Acad Sci USA.

[12] Nakamura T (2004). WHO diagnostic criteria for osteoporosis and trends in Europe and USA. Nihon Rinsho.

[13] Mazziotti G, Formenti A, Adler R, Bilezikian JP, Grossman A, Sbardella E (2016). Glucocorticoid-induced osteoporosis:pathophysiological role of GH/IGF-I and PTH / VITAMIN D axes, treatment options and guidelines. Endocrine.

[14] Gao L, Gao HZ, Ren Y (2014). The clinical observation of bone metabolic index and cytokine IGF-1 in postmenopausal osteoporosis patients. Chin J Lab Diagn.

[15] Xian LL, Wu XW, Pang LJ, Lou M, Rosen CJ, Qiu T (2012). Matrix IGF-1 maintains bone mass by activation of mTOR in mesenchymal stem cells. Nat Med.

[16] Ding L, Li X, Sun H, Su J, Lin N, Péault B (2014). Transplantation of bone marrow mesenchymal stem cells on collagen scaffolds for the functional regeneration of injured rat uterus. Biomaterials.

[17] Huang JY, Zhao YL, Li YF (2015). Correlation of osteoporosis and matrix metalloproteinases. J Hubei Univer Chin Med.

[18] Ogo Y, Taniuchi S, Ojima F, Hayashi S, Murakami I, Saito Y (2014). IGF-1 gene expression is differentially regulated by estrogen receptors αand βin mouse endometrial stromal cells and ovarian granulosa cells. J Reprod Dev.

[19] Chrobak I, Lenna S, Stawski L, Trojanowska M (2013). Interferon-γpromotes vascular remodeling in human microvascular endothelial cells by up-regulating endothelin (ET)-1 and transforming growth factor(TGF) β2. J Cell Physiol.

[20] Ljunghall S, Johansson AG, Burman P, Kämpe O, Lindh E, Karlsson FA (2010). Low plasma levels of insulin-like growth factor 1 (IGF-1) in male patients with idiopathic osteoporosis. J Intern Med.

[21] Muratli HH, Çelebi L, Hapa O, Biçimoğlu A (2005). Comparison of plasma endothelin levels between osteoporotic, osteopenic and normal subjects. BMC Musculoskelet Disord.

[22] Reed BY, Zerwekh JE, Sakhaee K, Breslau NA, Gottschalk F, Pak CY (2010). Serum IGF 1 is low and correlated with osteoblastic surface in idiopathic osteoporosis. J Bone Miner Res.

